# Desmoid-type Fibromatosis of the Breast Mimicking Carcinoma

**DOI:** 10.5334/jbsr.1612

**Published:** 2019-01-29

**Authors:** Laura Wuyts, Aljosja De Schepper

**Affiliations:** 1 GZA Hospitals, BE

**Keywords:** Mammary fibromatosis, desmoid fibromatosis, desmoid tumor, extra-abdominal desmoid tumor, aggressive fibromatosis

## Case

A 43-year-old female with left-sided mastodynia was referred to our department for magnetic resonance imaging (MRI). Her surgical history included an excisional biopsy of a fibroadenoma in the left breast.

**Figure 1:**
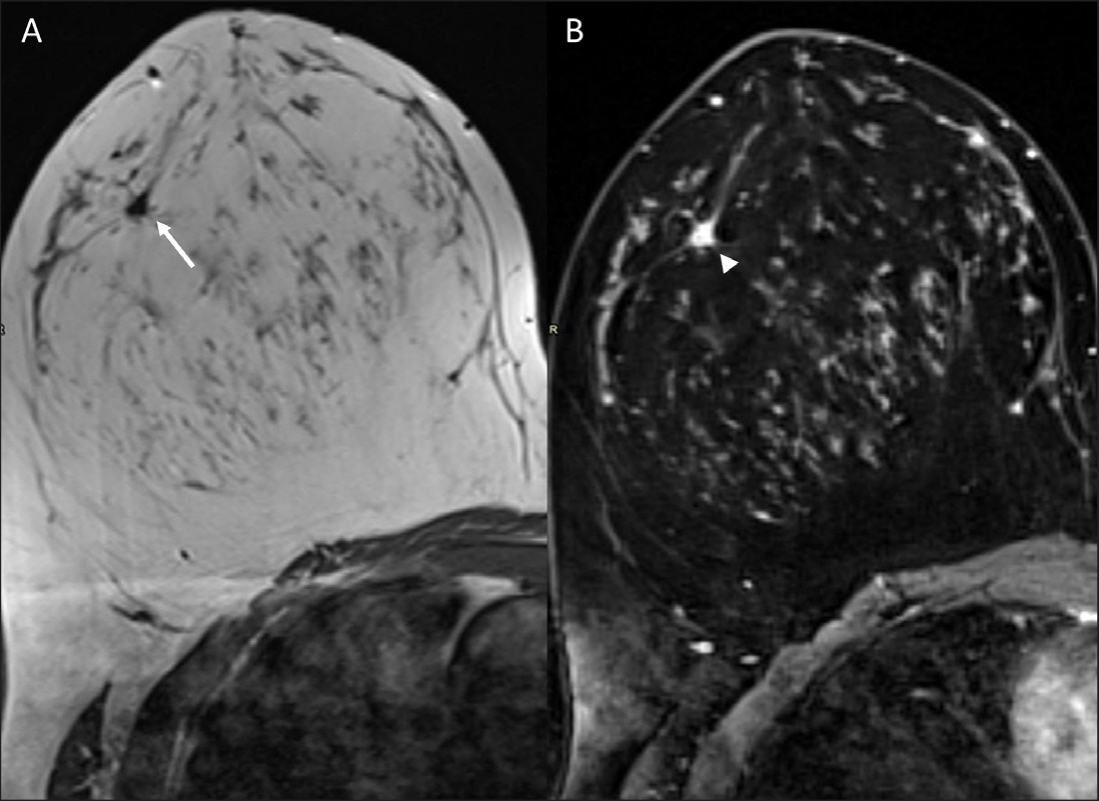
**(A)** Axial T1-weighted and **(B)** axial T1-weighted fat-suppressed contrast-enhanced MR images. Desmoid tumor superolateral in the right breast with irregular and stellate configuration (arrow) and enhancement (arrowhead).

On MRI, no explanation for the clinical complaints could be found. However, an enhancing stellate lesion (7 mm) at 9 o’clock in the contralateral right breast was noted (Figure 1B, arrowhead). The lesion had a homogeneously low signal on T1-weighted images (Figure 1A, arrow) and rapid early enhancement with plateau kinetics on dynamic contrast imaging. There was no diffusion restriction. Because of the discrepancy between the suspicious morphological findings and the rather reassuring dynamic characteristics, further mammography and breast ultrasound evaluation were recommended.

**Figure 2:**
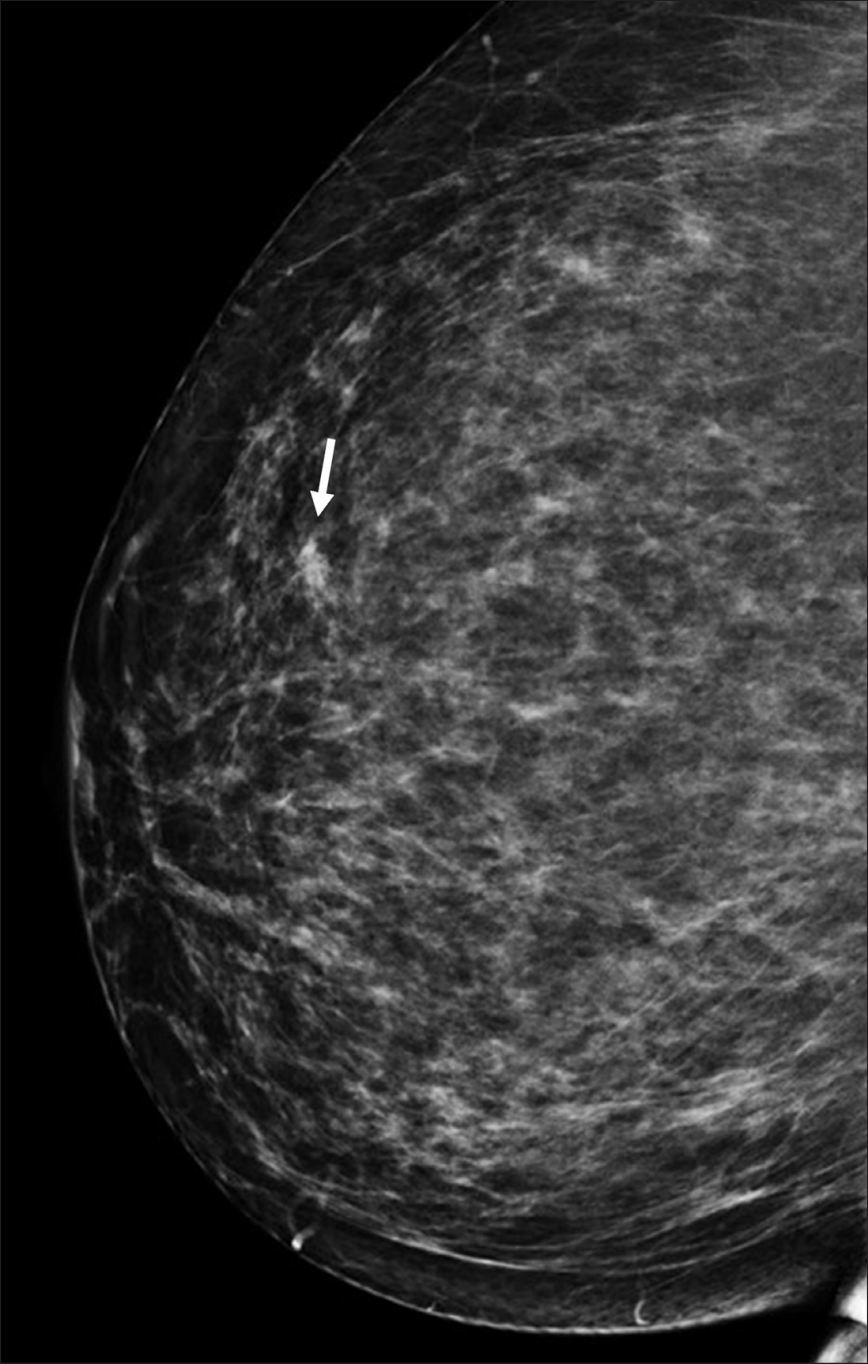
Right craniocaudal mammogram. Desmoid tumor with spiculated margins (arrow) in the upper-outer quadrant. No associated microcalcifications were identified.

**Figure 3:**
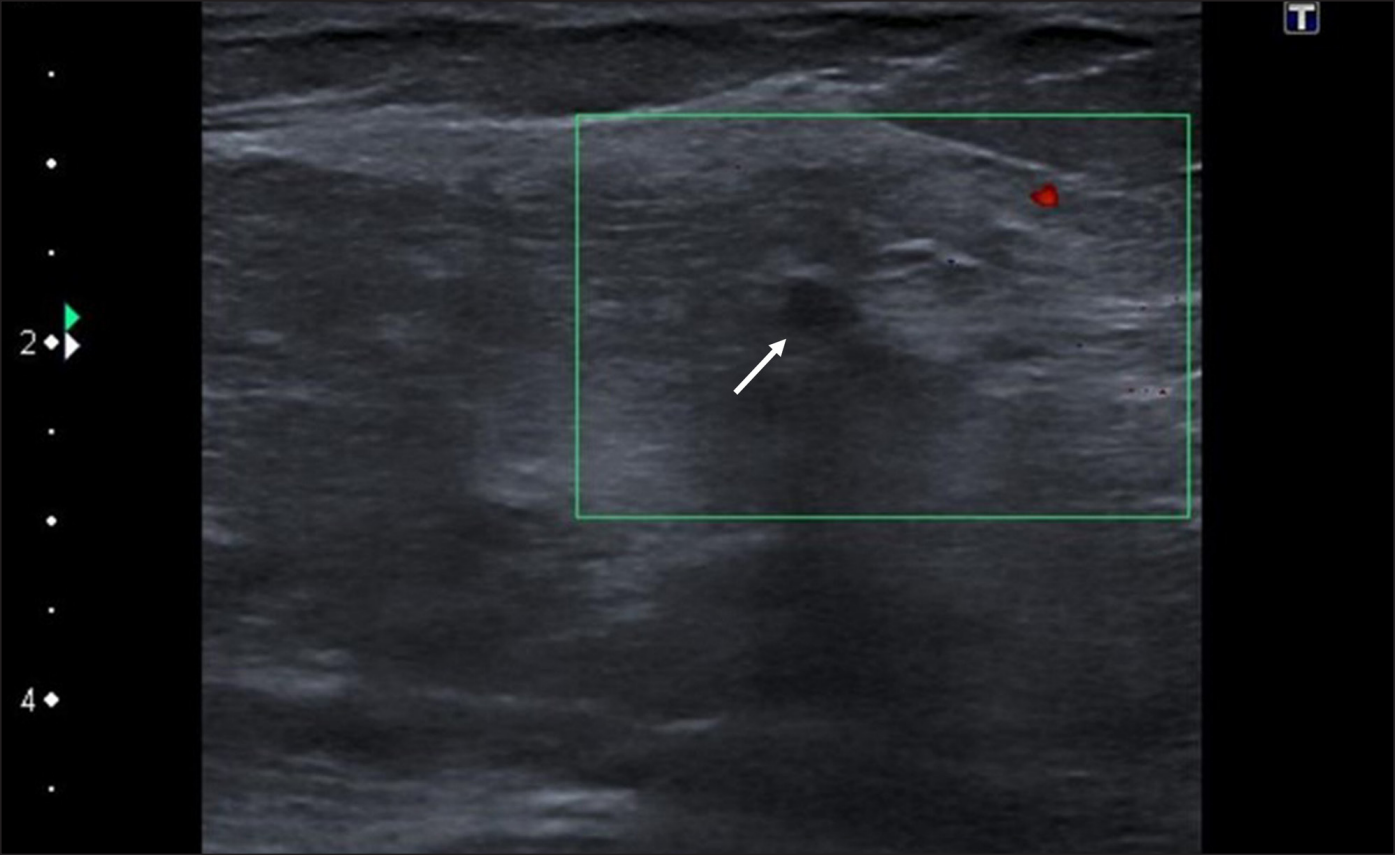
Color Doppler ultrasound. Breast ultrasound of the non-palpable was lesion demonstrated a solid, hypo-echoic mass (arrow) with partially obscured borders. No internal vascularization is identified.

On mammography of the right breast, a focal asymmetric density with spiculated margins was seen in the superolateral quadrant (Figure 2, arrow). There were no associated microcalcifications or suspicious lesions elsewhere. A second-look ultrasound in this region demonstrated a small oval hypoechoic mass (4 mm) with partially obscured borders (Figure 3, arrow). This lesion was categorized as a BI-RADS 4b lesion, and core needle biopsy was advised.

Histological analysis revealed an infiltrative stromal process with interlacing bundles of fibroblast and myofibroblast, suggestive for a desmoid tumor. No nuclear atypia or mitosis were seen. Wide excision was recommended and confirmed fibromatosis.

## Comment

A desmoid tumor or fibromatosis of the breast is a rare benign stromal tumor. Despite its benign nature and lack of metastatic potential, the tumor can be locally infiltrative. It mostly affects women during their reproductive years, often following trauma or various surgical breast procedures. In the literature, occasional association with familial adenomatous polyposis syndrome is reported. As with our case, no underlying cause was identified.

A desmoid tumor can occur anywhere in the breast, but tends to be in close proximity to the pectoralis major muscle, arising from the pectoral fascia. Patients can be asymptomatic or present with a painless and firm mass. The lump may be fixed to the chest wall if originating from the pectoral fascia. Occasionally, associated skin thickening and retraction is noted.

Imaging characteristics are non-specific. On ultrasound, mammography and MRI, fibromatosis manifests as a suspicious solid mass with irregular and spiculated borders, making it difficult to differentiate from primary breast malignancy. There is no lymphadenopathy, and microcalcifications on mammography are rare. Treatment consists of wide local excision, considering the high local recurrence rate after incomplete surgical resection.

## Conclusion

Desmoid-type fibromatosis is a mimicker of malignancy, with suspicious morphological findings on all imaging modalities due to the local infiltrative characteristics. Dynamic features on MR imaging may suggest a benign entity, but nonetheless, a biopsy remains the appropriate approach to rule out a primary breast tumor.

